# A review of multiple sclerosis: From pathophysiology to latest therapeutic advances

**DOI:** 10.3934/Neuroscience.2025026

**Published:** 2025-10-31

**Authors:** Arosh S. Perera Molligoda Arachchige, Jad El Choueiri, Francesca Pellicanò, Francesco Laurelli, Gabriel Amorim Moreira Alves, Niccolò Stomeo

**Affiliations:** 1 Faculty of Medicine & Surgery, University of Milan, Via Festa del Perdono 7, 20122 Milano MI, Lombardy, Italy; 2 Humanitas University, Via Rita Levi Montalcini 4, Pieve Emanuele 20072, Lombardy, Italy

**Keywords:** multiple sclerosis, autoimmune disease, central nervous system, demyelination, neuroinflammation, disease-modifying therapies, magnetic resonance imaging, neuroprotection, Bruton's tyrosine kinase inhibitors, stem cell therapy, McDonald criteria

## Abstract

Multiple sclerosis (MS) is a chronic autoimmune disorder characterized by inflammation, demyelination, and neurodegeneration within the central nervous system (CNS). It predominantly affects women and young adults, with environmental and genetic factors contributing to its onset. MS presents a wide range of neurological symptoms due to the scattering of lesions in the CNS, often leading to vision, sensorimotor, and cognitive impairments. The clinical course of MS varies, with relapsing-remitting MS (RRMS) being the most common, followed by secondary progressive MS (SPMS) and primary progressive MS (PPMS). Diagnosis is based on clinical evaluation, MRI findings, and cerebrospinal fluid analysis, with the McDonald criteria playing a key role in confirming dissemination in time and space. Current treatments, such as disease-modifying therapies (DMTs) and steroids, focus on managing relapses and reducing long-term disability. Novel therapies, including remyelination and neuroprotective agents, are showing promise in advancing care. While these medications can slow progression and improve quality of life, MS remains an incurable disease that requires ongoing research to find more effective therapies. Surgical interventions are rare but can address severe symptoms like spasticity and bladder dysfunction, contributing to an overall personalized management approach.

## 1. Introduction

Multiple sclerosis (MS) is a chronic inflammatory demyelinating disease that selectively affects the central nervous system (CNS). It is a primary disease of the CNS [1]. The pathological hallmark is focal demyelination, forming focal lesions known as plaques within the brain, spinal cord, and optic nerves. Since oligodendrocytes are not always able to restore the myelin, astrocytes produce gliotic plaques, a type of scars that cause a block in the transmission as secondary axonal damage [2],[3]. Because of the pathological association with demyelination and inflammation, MS is considered an autoimmune disease [4]. To date, the precise trigger of MS remains unknown; however, it is well established that the disease involves immune system activation. Although MS is not primarily an antibody-mediated condition, disease-modifying therapies (DMTs) that modulate the immune response have been shown to reduce the frequency and severity of relapses and may slow disability progression.

This review aims to provide a state-of-the-art synthesis by integrating the 2024 revision of the McDonald criteria, to summarize cutting-edge disease-modifying strategies such as Bruton's tyrosine kinase inhibitors and the emerging AMIGO-3 remyelination target, and to critically examine how these advances are reshaping both diagnosis and treatment paradigms.

## 2. Epidemiology

MS is a chronic autoimmune disorder that disproportionately affects women, with females about 2–3 times more likely than males to develop the disease. It typically begins between ages 20 and 40 and is one of the most common neurological conditions in this age group. Pediatric-onset MS accounts for roughly 2% of all cases, and only about 5% of new diagnoses occur after age 50 [5]–[7]. Multiple sclerosis (MS) affects more than 2.8 million people worldwide, with the highest prevalence in northern Europe, North America, and parts of Australasia, and a rising incidence in Asia, the Middle East, and Latin America. Within this global landscape, Italy reports among the highest prevalence rates in southern Europe—around 20–25 cases per 100,000 population—with an annual incidence of about 3 per 100,000 (≈2000 new diagnoses each year). Prevalence is higher in the north than in the south, a pattern partly linked to sun exposure and vitamin D status in combination with genetic susceptibility [8],[9].

## 3. Etiopathogenesis

**Figure 1.**
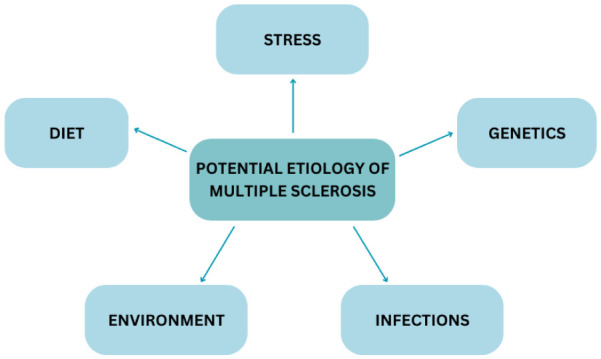
Diagram showing the potential etiopathogenesis of multiple sclerosis.

Despite extensive research, the causes of MS remain unknown. Like other autoimmune diseases, MS is believed to be caused by a combination of genetic susceptibility, abnormalities in the immune system, and infections (Figure 1) [10]. Environmental factors play a significant role in the development and progression of MS. Smoking has been consistently associated with an increased risk of developing MS and may also accelerate disease progression by promoting chronic inflammation and neurodegeneration. Climate, for instance, has been linked to MS prevalence, particularly in regions with limited sun exposure, which leads to vitamin D deficiency [11],[12]. Insufficient vitamin D, which is synthesized in the skin after sun exposure, may play a key role in the development of MS due to its influence on the immune system [11],[12]. Vitamin D3, specifically its receptor, plays an important role in immune function and is present on T regulatory cells, which are crucial in maintaining immune system balance [13]. An epidemiological study on the Jewish population in Israel revealed an interesting trend: individuals who moved to Israel before the age of 14 adopted the country's MS risk, while those who moved after the age of 15 retained the risk levels associated with their country of origin [14]. This finding suggests that early environmental exposure may have a significant impact on MS susceptibility. While dietary factors such as gluten-free diets and the role of vaccines have been discussed in the context of MS, there is currently no conclusive evidence supporting their impact on disease onset or progression. On the other hand, some studies suggest that physical and emotional stress may influence symptom exacerbation, although this relationship is not fully understood and remains an area of ongoing investigation [15],[16]. Infections, particularly with the Epstein–Barr virus (EBV), have been associated with MS. Around 99% of MS patients test positive for EBV antibodies, but not all individuals with EBV antibodies develop MS, indicating that genetic predisposition is necessary for the disease to manifest [17]. This pattern is similar to another autoimmune condition, Guillain-Barré syndrome, where a prior infection with *Campylobacter jejuni* increases the risk of the syndrome, though genetic factors also play a role in determining which individuals will develop the disease [18]. Genetics also significantly contributes to MS susceptibility. Twin studies have provided some of the most compelling evidence for the genetic link. Identical twins, who share the same genetic makeup, have a 25% risk of developing MS, which increases to 40% among homozygotic twins [19]. This is a stark contrast to the 0.1%–0.2% risk in the general population. Additionally, around 20% of people with MS have a blood relative who also has the disease, reinforcing the idea that a genetic predisposition exists. Studies have identified specific genetic markers, such as the HLA–DR2 haplotype (DRB1 1501–0602) and HLA–DR15, which are associated with earlier onset in females, as well as HLA–DQ6, all of which are linked to an increased likelihood of developing MS [20]. In conclusion, while environmental and genetic factors both contribute to MS, the precise trigger for the autoimmune response against myelin remains unknown. Some sources attribute this to autoreactive T cells, which get activated in the periphery and cross the blood–brain barrier, initiating an inflammatory response within the CNS and leading to demyelination, axonal injury, and neurodegeneration. B cells also play a key role by acting as antigen-presenting cells and producing pro-inflammatory cytokines, though the exact autoantigen remains unidentified [21]. The unpredictable nature of MS attacks adds further complexity to the disease, with some patients experiencing relapses after several years and others suffering more frequent attacks. The progression of MS can vary widely, with some individuals becoming wheelchair-bound within a year, while others may never reach that stage.

## 4. Clinical presentation

In MS, lesions are scattered throughout the CNS and tend to concentrate in specific regions, leading to a wide range of neurological symptoms [22]. One of the primary areas affected is the optic nerve, often resulting in visual disturbances such as blurred vision, double vision, or even temporary loss of vision in one eye. Lesions in the subpial spinal cord, particularly along the long pathways like the pyramidal tract and sensory tract, can cause motor and sensory deficits, including muscle weakness and numbness. The brainstem is another common site for MS lesions, leading to issues with balance, coordination, and even speech [23]. Involvement of the cerebellum further exacerbates coordination difficulties, often manifesting as tremors or instability. Lesions can also be found in both the cortical and juxtacortical regions, potentially affecting higher cognitive functions, memory, and reasoning. Finally, the periventricular white matter regions, located near the brain's ventricles, are frequently impacted, which is a hallmark of MS and contributes to the disease's characteristic dissemination across multiple CNS sites [24]. These diverse lesion locations explain the variability of symptoms seen in MS patients, as different regions of the CNS control various motor, sensory, and cognitive functions. The widespread nature of the lesions also makes MS a challenging disease to predict and manage, as symptoms can evolve and worsen over time depending on which areas of the CNS are affected.

**Figure 2.**
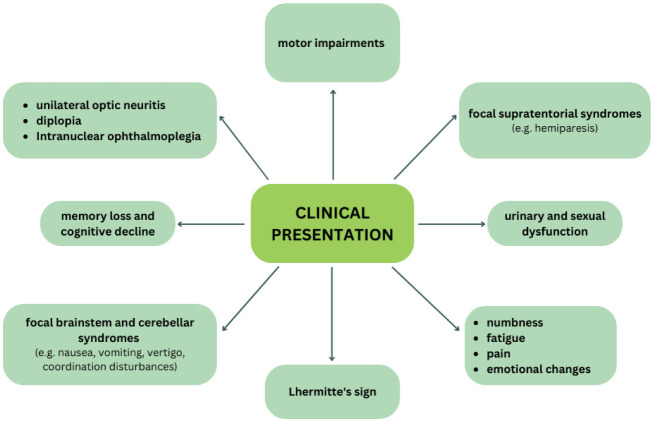
Diagram showing the clinical presentation of multiple sclerosis.

In MS, the disease often begins with a sudden attack, known as a relapse or exacerbation, during which patients may experience significant neurological impairments, such as being unable to walk. After this initial episode, they often recover and return to a relatively normal state. However, after a variable period, a second attack may occur, marking the beginning of a relapsing-remitting course of MS [25]. A relapse in MS is defined as a monophasic clinical episode where the patient reports neurological symptoms, and objective findings confirm a focal or multifocal inflammatory demyelinating event in the CNS [26]. These relapses develop acutely or sub-acutely and last for at least 24 hours, occurring without any concurrent fever or infection [27]. The specific symptoms experienced during a relapse depend on the location of the lesions within the CNS. Common initial manifestations of MS include unilateral optic neuritis, which causes vision problems in one eye and, in some cases, diplopia (double vision) if the third, fourth, or sixth cranial nerves are impaired [28],[29]. Intranuclear ophthalmoplegia can also occur [30]. Patients may also present with focal supratentorial syndromes, such as hemiparesis (weakness on one side of the body), or focal brainstem and cerebellar syndromes, leading to symptoms like nausea, vomiting, vertigo, and coordination disturbances [31]. When the spinal cord is involved, known as partial myelopathy, patients may experience paraparesis, sensory or motor impairments, and urinary dysfunction such as urgency or retention. Common symptoms during MS relapses include vision problems in one eye, numbness, difficulty walking, fatigue, emotional changes, vertigo, and dizziness. As the disease progresses, cognitive changes such as memory loss and cognitive decline can occur. Other potential symptoms include sexual dysfunction, balance and coordination issues, spasticity, bowel and bladder dysfunction, and pain. Some patients may also develop trigeminal neuralgia or paroxysmal dysarthria due to demyelination of the trigeminal nerve at its entry point in the pons, or hemifacial spasm from demyelination of the facial nerve at the midbrain. Additionally, Lhermitte's sign, a sudden sensation of electric shocks down the spine when bending the neck, may be present, indicating spinal cord involvement [32],[33]. The variability in the location and extent of lesions in MS explains the wide range of symptoms patients experience, making the disease unpredictable and its course difficult to forecast (Figure 2).

## 5. Clinical course of the disease

Multiple sclerosis is currently classified into clinical phenotypes based on disease course and activity, as proposed by Lublin et al. These include clinically isolated syndrome (CIS), relapsing-remitting MS (RRMS), primary progressive MS (PPMS), and secondary progressive MS (SPMS). Each phenotype can be further characterized by disease activity (active vs. not active) and progression (with vs. without progression), allowing a more nuanced description of individual disease trajectories [34].

### 5.1. Clinical isolated syndrome (CIS)

CIS refers to the first episode of neurological symptoms caused by inflammation and demyelination in the central nervous system, which is suggestive of MS. CIS often brings young patients to the hospital, with optic neuritis being a common presentation [35]. While some individuals with CIS go on to develop relapsing-remitting MS (RRMS), others do not experience further demyelinating episodes. Studies indicate that the likelihood of a second attack is significantly higher (88%) if brain lesions are detected on the initial MRI. Conversely, the risk drops to 19% if the MRI is normal [36].

### 5.2. Radiologically isolated syndrome (RIS)

RIS occurs when someone has magnetic resonance imaging (MRI) scans showing brain or spinal cord lesions characteristic of multiple sclerosis (MS), but has no neurological symptoms of MS. Some individuals with RIS will develop symptomatic MS over time, making RIS a potential early, or presymptomatic, stage of the disease. Risk factors such as younger age, male sex, certain MRI findings, and spinal cord lesions can increase the likelihood of developing MS from RIS [37].

### 5.3. Relapsing-remitting MS (RRMS)

This is the most common form of MS, affecting 85% of newly diagnosed patients. RRMS typically occurs in younger individuals and is characterized by episodes of relapse followed by periods of remission, where symptoms may improve or even disappear completely. After the first episode, patients experience remission, followed by relapses that cause further disability. While patients may be neurologically stable between relapses, over time, recovery becomes incomplete, leading to the accumulation of permanent disability after several relapses [38].

### 5.4. Secondary-progressive MS (SPMS)

SPMS begins as RRMS, with relapses and remissions, but eventually transitions into a progressive form. In SPMS, the patient experiences a steady worsening of neurological function independent of relapses. This progressive decline is marked by increasing disability that no longer follows the pattern of relapse and remission seen in RRMS [39].

### 5.5. Primary-progressive MS (PPMS)

Approximately 10% of MS patients have PPMS, a form characterized by continuous neurological decline from the onset without distinct relapses [40]. PPMS generally has a slower onset, but patients experience a gradual worsening of their condition over time. Some patients with PPMS may experience occasional relapses or new MRI activity superimposed on the progressive course. This phenotype, previously referred to as progressive-relapsing MS (PRMS), is now classified within PPMS with active disease [41]. Currently, Ocrelizumab, a monoclonal antibody targeting CD-20, is the only approved treatment shown to reduce disability progression in PPMS [41].

## 6. Diagnosis according to the 2024 Revised McDonald criteria

The diagnosis of MS relies on a combination of clinical evaluation, magnetic resonance imaging (MRI), and cerebrospinal fluid (CSF) analysis. The 2024 Revised McDonald criteria, developed by an international expert panel and presented at the ECTRIMS 2024 by Xavier Montalban, introduce several updates to enhance diagnostic accuracy while preserving specificity. The revised framework maintains the foundational principles of dissemination in space (DIS) and dissemination in time (DIT), while refining their definitions and incorporating new paraclinical tools [42],[43].

DIS now includes five topographic CNS regions: periventricular, cortical/juxtacortical, infratentorial, spinal cord, and the newly added optic nerve. Evidence of an optic nerve lesion—symptomatic or asymptomatic—can be supported by orbital MRI, visual evoked potentials (VEP), or optical coherence tomography (OCT), provided it aligns with a typical clinical presentation such as unilateral optic neuritis [44],[45].

DIT can be established by the presence of both enhancing and non-enhancing lesions on a single scan, the appearance of new lesions on follow-up MRI, or by CSF biomarkers. In addition to oligoclonal bands (OCBs), the kappa-free light chain index has been introduced as an alternative CSF marker for intrathecal B-cell activation, fulfilling DIT criteria with approximately 87% concordance to OCBs [45].

Moreover, the 2024 criteria incorporate advanced MRI biomarkers, including the central vein sign (CVS) and paramagnetic rim lesions (PRLs) (see Box 1). The CVS reflects perivenular inflammation, with high specificity for MS, while PRLs indicate chronic active lesions and may signal disease progression. These imaging features, particularly on susceptibility-weighted sequences such as FLAIR* or T2*, serve to support diagnosis in patients with typical presentations and reduce misdiagnosis in ambiguous cases [43].

Importantly, the revised criteria emphasize their application in typical clinical syndromes, such as unilateral optic neuritis, partial myelopathy, and focal brainstem or cerebellar syndromes, and advise against their use in atypical presentations (e.g., bilateral optic neuritis, encephalopathy, or isolated fatigue) [43],[44].


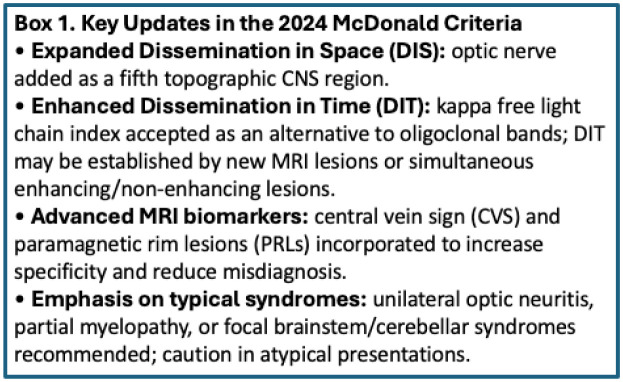


### 6.1. MRI findings in multiple sclerosis (updated according to 2021 MAGNIMS–CMSC–NAIMS guidelines)

MRI remains a cornerstone in the diagnosis, prognosis, and monitoring of MS. The 2021 MAGNIMS–CMSC–NAIMS guidelines emphasize the use of standardized MRI protocols, including 3D FLAIR sequences as the core sequence for brain imaging due to their high sensitivity for detecting lesions and enabling accurate comparisons over time [45].

*Typical MRI findings:*

• T2-hyperintense lesions: Reflect areas of demyelination and gliosis, appearing bright on T2-weighted images.

• T1-hypointense lesions (so-called “black holes”): Indicate more severe axonal damage and permanent tissue loss.

• Gadolinium-enhancing lesions: Denote active inflammation and are critical for demonstrating dissemination in time (DIT).

**Figure 3.**
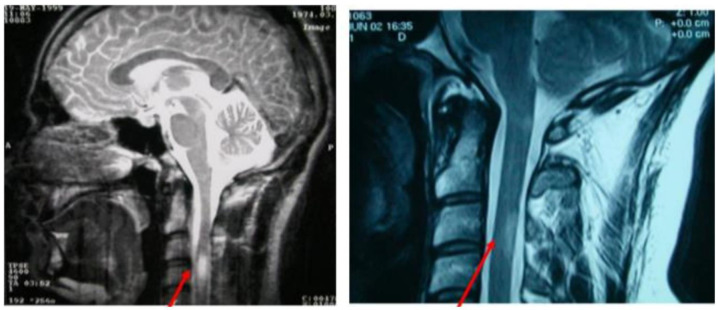
Spinal cord lesions on MRI.

**Figure 4.**
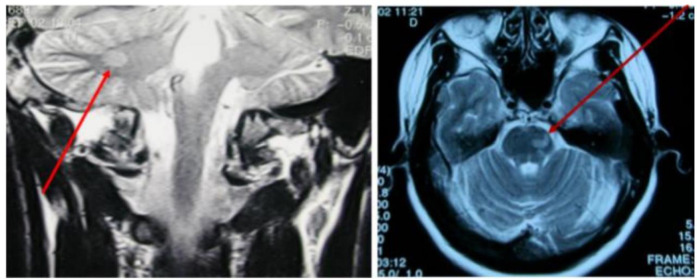
Infratentorial lesions on MRI.

**Figure 5.**
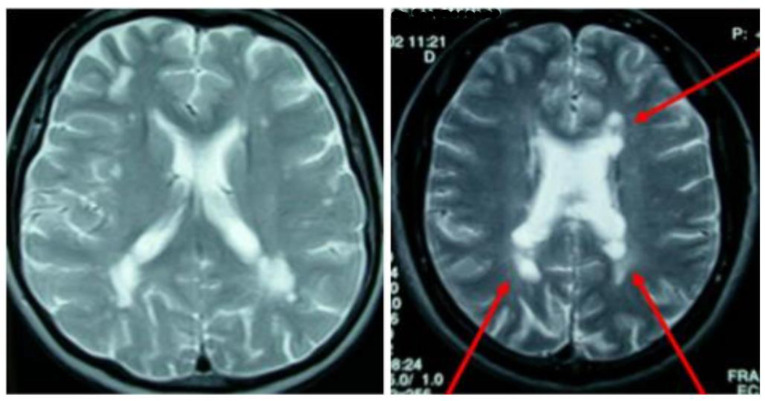
Periventricular lesions on MRI.

**Figure 6.**
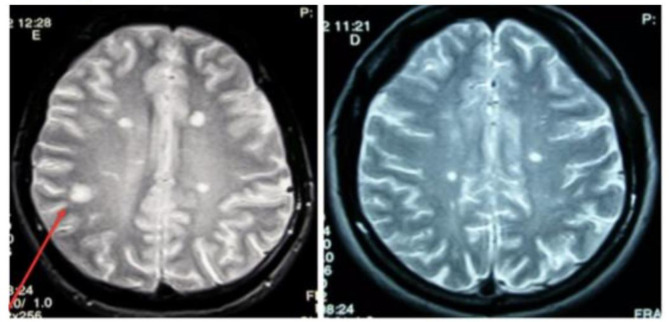
Juxtacortical lesion on MRI.

**Figure 7.**
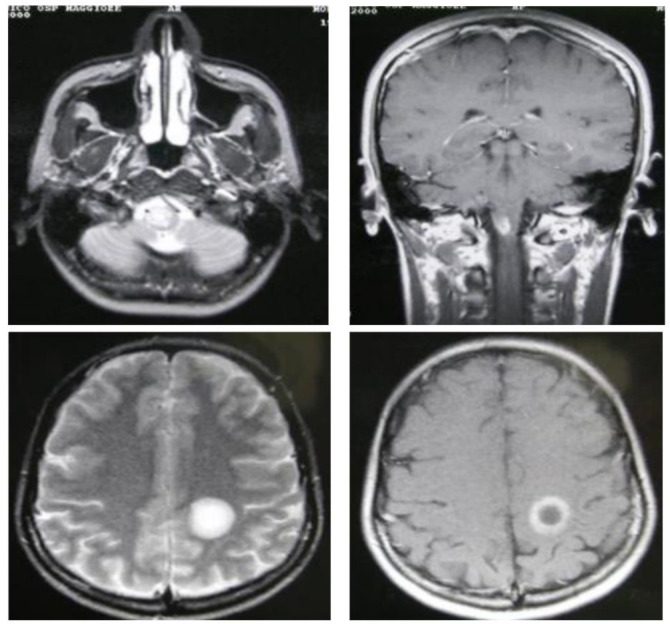
T1-weighted lesions enhanced by gadolinium.

Dissemination in space (DIS) can be demonstrated by T2-hyperintense lesions in at least two of four characteristic CNS regions (periventricular, cortical/juxtacortical, infratentorial, and spinal cord). DIT is shown by the simultaneous presence of enhancing and non-enhancing lesions or by new lesions on follow-up MRI, without requiring contrast if previous imaging is available for comparison (Figures 3–7, Table 1) [45].

*Protocol enhancements:*

• Use of 3D T2-weighted FLAIR (ideally at 3T) is preferred over 2D sequences due to improved lesion detection, especially in the posterior fossa and cortex.

• Spinal cord MRI is emphasized for both diagnostic and prognostic value, especially in patients with clinically isolated syndrome (CIS) or progressive MS.

• Gadolinium-based contrast agents (GBCAs) are recommended for initial diagnostic scans but their use in follow-up is minimized unless clinical decisions depend on demonstrating new activity or differential diagnosis [45].

*Key updates from 2021:*

• 3D FLAIR is the core sequence for brain MRI in MS diagnosis.

• Gadolinium is no longer routinely required for demonstrating DIT on follow-up, provided a baseline is available.

• Spinal cord imaging should cover the cervical and upper thoracic segments (C1 to T5) as a compromise between sensitivity and scan time.

• Advanced techniques (e.g., PSIR, central vein sign, cortical lesion detection) are optional and recommended only in centers with appropriate expertise [43].

**Table 1.** MRI acquisition recommendations for diagnosing, monitoring, and ensuring the safety of patients with multiple sclerosis, based on the 2021 MAGNIMS–CMSC–NAIMS consensus guidelines.

Region	Purpose	Recommended sequences	Field strength	Slice thickness	In-plane resolution	Contrast use	Notes
Brain	Diagnosis (DIS/DIT)	Sagittal 3D FLAIR (core)-Axial T2-weighted TSE-Axial/3D post-contrast T1-weighted	≥1.5T (preferably 3T)	3D: 1 mm isotropic 2D: ≤3 mm, no gap	≤1 mm × 1 mm	Gadolinium recommended at baseline	Core protocol for initial diagnosis. Use identical sequences in follow-up.
	Monitoring	Sagittal 3D FLAIR-Optional T2 axial-Post-contrast T1 only if clinical need	≥1.5T	As above	As above	Optional	Avoid contrast if a comparable recent MRI is available.
	Safety (e.g., PML)	FLAIR-T2-weighted-DWI-Optional T1 post-contrast	≥1.5T	3D or 2D ≤3 mm	≤1 mm × 1 mm	Recommended if suspicious lesion seen	PML screening every 3–4 months in high-risk patients.
Spinal cord	Diagnosis	Sagittal T2-weighted TSE or STIR. Optional: Proton density-weighted-Post-contrast sagittal T1-weighted	≥1.5T (3T not proven superior)	Sagittal: ≤3 mm, no gap Axial: ≤5 mm, no gap	≤1 mm × 1 mm	Optional (recommended if brain MRI is contrast-enhanced)	Coverage should include C1–T5 at minimum. Use axial images to clarify uncertain lesions.
	Monitoring	Same as diagnosis (only if clinically indicated)	≥1.5T	As above	As above	Optional	Not routinely recommended unless symptoms or unexplained disability progression.
	Safety	Not applicable	–	–	–	Not required	Spinal cord MRI not used for safety monitoring.
Optic nerve	Diagnosis (selective)	Axial & coronal fat-suppressed T2/STIR-Post-contrast T1-weighted (optional)	≥1.5T	≤2–3 mm, no gap	≤1 mm × 1 mm	Optional	Use only if atypical optic neuritis, pediatric optic neuritis, or for differential diagnosis.
	Monitoring	Only if new or progressive visual symptoms	≥1.5T	As above	As above	Optional	Interpret with clinical findings and visual evoked potentials.

Note: Key technical considerations include the use of ≥1.5T field strength (preferably 3T for brain imaging), standardized slice thickness (≤3 mm for 2D and 1 mm isotropic for 3D sequences), and in-plane resolution of ≤1 mm × 1 mm. For gadolinium-based contrast agent (GBCA) use, macrocyclic agents at 0.1 mmol/kg body weight are advised with a post-injection delay of at least 5–10 minutes before T1-weighted imaging. Imaging protocols must remain consistent across timepoints with respect to scanner, sequence type, spatial resolution, and positioning. While 3D FLAIR is the preferred core brain sequence, spinal cord imaging should cover at least the C1–T5 segments. Advanced sequences such as DIR, PSIR, and susceptibility-weighted imaging (e.g., central vein sign) are optional and require local expertise. Routine spinal cord and optic nerve imaging are not required unless clinically indicated.

#### 6.1.1. Advanced quantitative MRI: Diffusion tensor imaging biomarkers in multiple sclerosis

In addition to conventional MRI sequences, diffusion tensor imaging (DTI) provides quantitative metrics that can serve as natural biomarkers of microstructural integrity in both white matter (WM) and gray matter (GM). Parameters such as fractional anisotropy (FA), mean diffusivity (MD), radial diffusivity (RD), and axial diffusivity (AD) allow early detection of tissue damage even in patients with minimal disability (low Expanded Disability Status Scale score values). Decreased FA and increased MD or RD in normal-appearing WM have been consistently associated with early axonal injury and demyelination, while similar abnormalities in cortical and deep GM (e.g., thalamus, basal ganglia) correlate with cognitive decline and fatigue, supporting DTI's role in detecting subclinical changes [46].

To ensure accuracy and reproducibility, DTI studies require rigorous preprocessing, including noise suppression, Gibbs ringing removal, bias field correction, and eddy current correction [47]. Advanced techniques such as B-matrix spatial distribution correction (BSD) and artificial intelligence-based correction (AIBSD) further improve the precision of DTI metrics by minimizing motion and distortion artifacts [48].

Emerging work demonstrates that DTI with systematic artifact correction enhances tractography quality and enables quantitative assessment of gray matter, expanding its prognostic potential beyond conventional WM analysis. Moreover, DTI-derived parameters have shown translational applicability across organ systems (e.g., hepatic fibrosis quantification), highlighting their methodological robustness and adaptability for neuroimaging research [49],[50].

Integrating DTI data with traditional MRI findings and clinical indicators such as EDSS may provide a comprehensive quantitative framework for monitoring disease progression, predicting treatment response, and evaluating neuroprotective or remyelinating therapies in both clinical and research contexts.

### 6.2. Cerebrospinal fluid (CSF) analysis

Before MRI became widely available, CSF analysis played a more prominent role in diagnosing MS. Today, CSF studies are still useful in supporting the diagnosis and ruling out differential diagnosis. CSF from MS patients typically shows mild pleocytosis in about 50% of cases, with white blood cell counts usually below 35 cells/mm³ [51]. More importantly, oligoclonal bands are found in 90%–95% of patients with definitive MS [52]. These bands reflect intrathecal synthesis of antibodies, indicating an abnormal immune response within the CNS. However, the presence of oligoclonal bands does not predict the course of the disease or its severity, as they remain consistent over time [53].

### 6.3. Evoked potentials (EPs)

These are diagnostic tests used to assess the conduction of nerve impulses to the brain, revealing delays that suggest demyelination. In MS, EPs are particularly useful when clinical examinations and MRI fail to provide clear evidence of lesion dissemination in space. They help uncover subclinical demyelinating lesions that may not be apparent through other methods [54].

Clinicians may consider obtaining evoked potentials when MRI or clinical findings are inconclusive. These tests can provide additional evidence of MS by detecting delays in nerve conduction.

(1) Visual evoked potentials (VEPs): VEPs measure the time it takes for visual stimuli to reach the visual cortex, and they are commonly delayed in MS patients due to prior demyelination of the optic nerve. This is often used to identify subclinical optic neuritis [55].

(2) Brainstem auditory evoked potentials (BAEPs): BAEPs measure nerve signals along the acoustic and brainstem pathways. They can detect lesions affecting these pathways, providing evidence of demyelination in the brainstem [56].

(3) Somatosensory evoked potentials (SSEPs): SSEPs assess the sensory pathways in the spinal cord and brain. Delayed signals in these pathways may indicate lesions along the spinal sensory tracts, helping to confirm MS diagnosis when other tests are inconclusive [57].

(4) Brainstem and spinal cord potentials: While less commonly abnormal in MS compared to VEPs, testing these pathways can still be useful in certain cases to detect demyelination along the brainstem and spinal cord [58].

## 7. Treatment

Early initiation of treatment is crucial in managing MS because, while current therapies cannot halt the disease entirely, disease-modifying therapies (DMTs) vary in their efficacy, with reductions in relapse rates ranging from approximately 30% with interferons to around 55% with fingolimod and up to 85% with natalizumab. This reduction in relapses helps to minimize long-term disability [59]. Current clinical guidelines, such as those from the European Committee for Treatment and Research in Multiple Sclerosis (ECTRIMS) and the European Academy of Neurology (EAN), as well as the American Academy of Neurology (AAN), recommend initiating treatment promptly after a confirmed diagnosis of relapsing MS to delay disease progression, reduce relapse rates, and minimize long-term disability. First, irreversible damage to axons occurs even in the early stages of the disease. Second, therapy is most effective during the early inflammatory phase of MS, and third, it is least effective once the disease has progressed to the neurodegenerative phase [60].

### 7.1. Treatment for acute attacks/relapses

During acute relapses, patients are typically treated with a course of steroids, such as intravenous methylprednisolone (1 g daily for 3–5 days, extending to 10 days if the relapse is severe). Steroids are anti-inflammatory agents that help shorten the duration of relapses, but they do not address the underlying cause of MS [61]. For patients with severe relapses who do not respond adequately to high-dose corticosteroids, therapeutic plasma exchange (PLEX) is recommended as a second-line treatment. According to the American Academy of Neurology (AAN) guidelines, PLEX can be effective in improving neurological outcomes, particularly in steroid-refractory cases. It works by removing circulating immune complexes and pathogenic autoantibodies implicated in demyelination.

### 7.2. Disease-modifying therapies (DMTs)

Several first-line treatments are available for relapsing-remitting multiple sclerosis (RRMS), including injectable options such as glatiramer acetate (GA), interferon beta-1a, and interferon beta-1b. These first-generation, low-efficacy disease-modifying therapies (DMTs) have modest effects on reducing relapse rates and disease progression [62]. GA is considered a particularly safe option during pregnancy [63],[64]. Oral medications such as teriflunomide and dimethyl fumarate are classified as platform therapies and are typically used in patients with mild to moderate RRMS. Fingolimod, the first oral sphingosine-1-phosphate (S1P) receptor modulator, is regarded as high-efficacy in most guidelines (though some still list it as a platform therapy) and carries risks such as bradycardia, atrioventricular block, and varicella-zoster virus reactivation [60],[64]. Siponimod is approved for active secondary progressive MS (SPMS), while cladribine (3.5 mg/kg), ozanimod, and ponesimod are oral high-efficacy agents approved for RRMS. Cladribine selectively depletes B and T cells with intermittent dosing, improving patient compliance and offering long-term disease control [65].

High-efficacy therapies are generally reserved for patients with highly active disease or inadequate response to platform therapies. These include monoclonal antibody infusions such as alemtuzumab, natalizumab, and the CD20 monoclonal antibodies (ocrelizumab, ofatumumab, and the recently approved ublituximab, which provides shorter infusion times and superior relapse-rate reduction versus teriflunomide). Notably, ocrelizumab remains the only DMT approved for primary progressive MS [66],[67].

Mitoxantrone, once used for aggressive RRMS and SPMS, is now rare because of cardiotoxicity and leukemia risk. In low-resource settings, off-label immunosuppressants such as azathioprine, cyclophosphamide, methotrexate, mycophenolate mofetil, or rituximab may be considered when approved DMTs are inaccessible (Tables 2 and 3) [68],[69].

**Table 2.** Common adverse effects associated with selected DMTs used in the treatment of multiple sclerosis, along with approximate frequencies.

Side effect	Associated medication(s)	Approximate frequency
Injection site reactions	Interferon beta, Glatiramer Acetate	Common (≥10%–60%)
Flu-like symptoms	Interferon beta	Very common (up to 70%)
Hepatotoxicity (↑ liver enzymes)	Interferon beta, Fingolimod, Teriflunomide, Natalizumab	Occasional to common (3%–15%)
Leukopenia/Lymphopenia	Fingolimod, Dimethyl Fumarate, Cladribine, Natalizumab	Common (15%–30%); Cladribine: Very common (up to 80%)
Thrombocytopenia	Cladribine	Less common (<10%)
Opportunistic infections	Fingolimod, Natalizumab, Cladribine	Rare to occasional; Cladribine: ~10%
Progressive multifocal leukoencephalopathy (PML)	Natalizumab, Fingolimod, Dimethyl Fumarate	Rare (<1/1000); Natalizumab: ~4/1000 in JCV + cases
Depression	Interferon beta	Occasional (~10%–15%)
Bradycardia/atrioventricular (AV) block	Fingolimod	Occasional (~5%)
Hair thinning	Teriflunomide	Common (~10%–13%)
Skin flushing/gastrointestinal upset	Dimethyl Fumarate	Very common (>30%)

Note: Frequencies are based on clinical trial data and post-marketing surveillance. “Very common” refers to adverse events occurring in >10% of patients; “common” in 1%–10%; “occasional” in 0.1%–1%; and “rare” in <0.1%. The risk of progressive multifocal leukoencephalopathy (PML) is highest with natalizumab, particularly in JC virus-positive individuals treated for over two years or with prior immunosuppressant use. Off-label medications and newer agents are not included in this table [70].

**Table 3.** Mode of administration, efficacy class, and approved clinical indications for commonly used disease-modifying therapies (DMTs) in multiple sclerosis.

Medication	Administration	Efficacy class	Approved indications
Interferon beta-1a/b	Injectable	Low	RRMS
Glatiramer acetate (GA)	Injectable	Low	RRMS
Teriflunomide	Oral	Moderate	RRMS
Dimethyl fumarate	Oral	Moderate	RRMS
Fingolimod	Oral	High	RRMS, Active SPMS
Siponimod	Oral	High	Active SPMS
Ozanimod	Oral	High	RRMS
Ponesimod	Oral	High	RRMS
Cladribine	Oral (short course)	High	RRMS, Active SPMS
Natalizumab	IV infusion	High	RRMS
Alemtuzumab	IV infusion	High	RRMS
Ocrelizumab	IV infusion	High	RRMS, PPMS
Ofatumumab	Subcutaneous	High	RRMS
Ublituximab	IV infusion	High	RRMS
Mitoxantrone	IV infusion	Moderate-high	Aggressive RRMS, SPMS (rarely used)
Rituximab (off-label)	IV infusion	High (off-label)	RRMS, PPMS (off-label)
Azathioprine, others	Oral/IV	Low (off-label)	RRMS (resource-limited settings)

Note: Indications include relapsing-remitting MS (RRMS), active secondary progressive MS (SPMS), and primary progressive MS (PPMS). Classification of efficacy is based on clinical trial data and current treatment guidelines (e.g., ECTRIMS, AAN, NICE). Off-label therapies such as rituximab and azathioprine may be used in specific clinical or resource-limited contexts.

### 7.3. Symptomatic treatment

In addition to disease-modifying therapies, symptomatic treatments are essential for managing multiple sclerosis (MS). Muscle spasticity is commonly treated with antispastic agents such as baclofen, tizanidine, and cannabinoids [71]. When oral medications prove insufficient for moderate to severe spasticity, intrathecal baclofen (ITB) pumps can be surgically implanted to deliver medication directly to the spinal cord, improving motor function and reducing systemic side effects [72],[73]. Despite its effectiveness, ITB therapy remains underutilized due to concerns over cost and the predominant focus on disease-modifying therapies [74].

Mood disorders associated with MS are typically managed with selective serotonin reuptake inhibitors (SSRIs) [71], while fatigue is often addressed with agents like amantadine or modafinil [71]. Sexual dysfunction can be treated pharmacologically with sildenafil [71]. Bladder dysfunction is commonly managed with anticholinergics such as oxybutynin; however, when conservative treatments fail, surgical options such as augmentation cystoplasty, which enlarges the bladder to restore low pressure and compliance, or non-continent urinary diversion using an ileal conduit and external urine collection device may be considered, particularly in wheelchair-bound or bedridden patients [75]–[78].

Pain management strategies include tricyclic antidepressants and cannabinoids, which help alleviate neuropathic pain [71]. Neurological symptoms like tremor may respond to beta-blockers and antiepileptic drugs [71], but for disabling tremors resistant to medication, deep brain stimulation (DBS) offers a surgical alternative. This procedure involves implanting electrodes in specific brain regions to regulate abnormal electrical signals, resulting in significant tremor reduction [79]–[81]. Orthopedic surgical interventions may also be necessary to address skeletal deformities and joint contractures arising from prolonged immobility, thereby improving mobility and comfort [82].

It is important to note that not all MS symptoms require specific treatment, especially mild sensory symptoms that often resolve spontaneously. However, symptoms that significantly impair daily functioning, such as visual disturbances or reduced mobility, usually necessitate medical or surgical management to improve the patient's quality of life. Surgical treatments do not alter the course of MS but are valuable for managing secondary complications unresponsive to pharmacological therapy. Surgical interventions should be carefully considered due to concerns about potential postoperative MS exacerbations; however, studies indicate no significantly increased relapse risk compared to non-MS patients [82]. A multidisciplinary and personalized approach is essential to minimize risks and optimize outcomes.

### 7.4. Novel treatment approaches

Recent advancements in MS research are revolutionizing treatment by addressing the disease at its core, moving beyond symptomatic management toward therapies that target immune dysfunction, neuroprotection, and remyelination.

**Figure 8.**
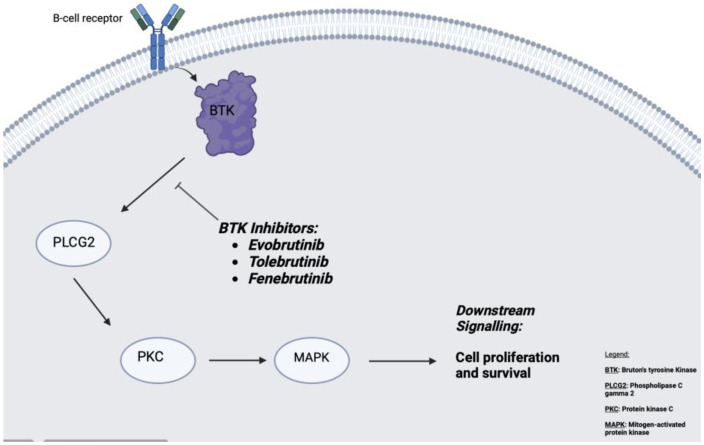
Diagram illustrating the mechanism of action of BTKi.

A significant shift in MS treatment is the development of Bruton's tyrosine kinase (BTK) inhibitors, such as evobrutinib and tolebrutinib, which target B cells and microglia. These oral therapies reduce inflammation by inhibiting BTK, offering a more precise approach than traditional immunosuppressants. Though these agents are still under clinical investigation, they represent a promising alternative to broad-spectrum therapies and may play a big role in both relapsing and progressive MS. Fenebrutinib, another BTK inhibitor currently in clinical trials, further expands this therapeutic class (Figure 8).

One experimental use of intrathecal mesenchymal stem cells (MSCs) aimed at reducing inflammation and promoting neuroprotection when administered directly into the cerebrospinal fluid. Although still in early clinical trials, MSCs show potential for enhancing remyelination and improving outcomes, particularly in progressive MS. However, evidence remains limited, and more robust trials are needed to confirm their safety and efficacy [83].

Opicinumab (anti-LINGO-1), another remyelination therapy, seeks to repair damaged myelin by inhibiting the LINGO-1 protein, which blocks myelination. Despite early-phase trials, such as the SYNERGY trial, failing to meet primary endpoints, they still managed to shift the attention of recent studies on the exact location and expression of LINGO-1 proteins [84].

The results led the scientific community to the discovery of the amphoterin-induced gene and open reading frame-3 (AMIGO-3) protein. Sharing similar characteristics with the LINGO-1, the former has been proposed as a better target for novel experimental drugs and is currently under deeper investigation [85].

In the area of remyelination, elezanumab, a monoclonal antibody targeting the regeneration-blocking repulsive guidance molecule A (RGMa) protein, aims to promote neural repair. This approach is distinct from currently approved immunomodulatory and anti-inflammatory treatments for both relapsing and progressive forms of MS. If proven effective in ongoing clinical trials, elezanumab could address unmet medical needs within the MS population [86]. Ibudilast, a phosphodiesterase inhibitor, offers neuroprotection by reducing inflammation and protecting neurons, showing promise especially for progressive MS, where treatment options remain limited [87].

Ibudilast, a phosphodiesterase inhibitor, focuses on neuroprotection by reducing inflammation and protecting neurons, showing promise for progressive forms of the disease where few effective treatments exist [87].

### 7.5. Practical considerations: Adherence, cost, and access

Beyond clinical trial efficacy, the long-term success of disease-modifying therapies (DMTs) hinges on adherence, affordability, and equitable access. Real-world studies show that up to 30% of patients discontinue or switch therapies within two years because of side effects, injection fatigue, or complex monitoring requirements [88]. High-efficacy monoclonal antibodies and S1P modulators, though effective, carry substantial acquisition and monitoring costs that can limit use, particularly in low- and middle-income settings. In many regions, delayed reimbursement, restricted formularies, and the need for specialized infusion facilities further constrain access. Addressing these practical barriers through patient education, simplified monitoring, and cost-containment strategies is critical to translating trial efficacy into durable population-level benefit.

## 8. Supportive care

### 8.1. Rehabilitation strategies in MS

Comprehensive rehabilitation is a key component of MS management and complements pharmacological therapy. Physiotherapy, including individualized exercise programs, balance training, and gait re-education, has been shown to improve mobility, reduce fatigue, and lower the risk of falls. Cognitive rehabilitation, through computer-assisted training or therapist-led exercises, targets deficits in memory, attention, and executive function, helping maintain independence and employment. Additional interventions such as occupational therapy, speech therapy, and energy-conservation techniques can be tailored to patient needs. Evidence supports a multidisciplinary approach, integrating neurologists, physiatrists, and therapists to optimize long-term outcomes [89].

### 8.2. Psychosocial consequences of MS

MS exerts profound psychosocial effects that extend beyond physical disability. Rates of depression and anxiety are two to three times higher than in the general population and can worsen fatigue, cognitive performance, and treatment adherence. Social and vocational impacts include loss of employment, financial strain, and challenges to intimate relationships and family planning. Early screening for mood disorders, timely referral to mental health services, patient support groups, and vocational counseling are recommended to preserve quality of life and social participation [90],[91].

## 9. Current controversies and knowledge gaps

Despite substantial progress, several core issues in MS remain unclear.

Vitamin D: Epidemiologic studies consistently associate low vitamin D status with increased MS risk, and supplementation is widely recommended for general health. However, randomized trials have not conclusively demonstrated that vitamin D supplementation prevents MS or alters long-term disability once the disease has developed, leaving its role as causal versus modulatory unresolved.

Epstein–Barr virus (EBV): Nearly all people with MS show prior EBV infection, and recent large cohort studies support EBV as a strong risk factor. Yet key questions remain, including whether EBV is a true cause, an essential trigger requiring additional cofactors, or simply a near-universal infection coincident with disease onset.

Stem cell therapies: Autologous hematopoietic stem cell transplantation and mesenchymal stem cell infusions offer promising immunoablative and neuroprotective effects. Nevertheless, controlled trials show variable efficacy, and safety concerns—including infection risk and treatment-related mortality—have slowed their adoption.

These examples illustrate how even well-studied aspects of MS pathobiology and therapy still contain major uncertainties. Ongoing mechanistic studies and long-term clinical trials will be essential to resolve these controversies and guide future treatment strategies [92],[93].

## 10. Conclusions

Together, these novel therapies and many others represent a shift toward a more targeted, personalized treatment that not only addresses disease progression but can also improve symptom management, thus significantly advancing the landscape of MS care. Beyond therapeutic innovations, the integration of advanced imaging biomarkers, particularly diffusion tensor imaging (DTI) metrics in white and gray matter, offers promising tools for quantifying microstructural integrity and detecting early, subclinical changes in MS.

A nuanced appraisal of current disease-modifying therapies (DMTs) underscores that efficacy and risk are not uniform. Injectable interferons and glatiramer acetate still show low efficacy but have excellent long-term safety and are particularly suitable for patients with mild disease or when safety during pregnancy is a priority. Moderate-efficacy oral agents such as teriflunomide and dimethyl fumarate balance convenience with manageable safety profiles. High-efficacy options—natalizumab, ocrelizumab, ofatumumab, alemtuzumab, cladribine, and S1P modulators like fingolimod or ozanimod—achieve up to 70%–85% relapse reduction but carry higher risks, including opportunistic infections (e.g., PML with natalizumab), autoimmune complications (e.g., thyroiditis with alemtuzumab), or cardiovascular effects (e.g., bradyarrhythmia with fingolimod). Careful patient selection and monitoring are therefore crucial.

Therapeutic timing is also evolving. Traditional “escalation” strategies—starting with low- to moderate-efficacy DMTs and switching only if the disease remains active—have the advantage of long-term safety data and lower upfront risk. However, an expanding body of evidence supports early highly effective treatment (EHT), in which potent DMTs are introduced soon after diagnosis to maximize early suppression of inflammation and possibly delay or prevent progressive phases. Head-to-head trials and long-term observational studies increasingly favor EHT for patients with poor prognostic factors (e.g., high lesion load, frequent early relapses), though concerns remain regarding cumulative immunosuppression and cost.

Taken together, these considerations reinforce that individualized therapy selection—balancing efficacy, safety, lifestyle factors, and patient preference—is key to optimizing long-term outcomes in multiple sclerosis.

Despite these therapeutic advances, major challenges remain. Most of the recently approved or experimental therapies act predominantly on the inflammatory, relapse-driven stage of multiple sclerosis, offering limited benefit in progressive, neurodegenerative phases. Translating neuroprotective and remyelinating approaches into clinical success has proven difficult, as illustrated by the failure of the anti-LINGO-1 antibody opicinumab and the still preliminary status of mesenchymal stem cell and Bruton's tyrosine kinase inhibitor programs. Furthermore, long-term safety profiles of many high-efficacy agents remain incompletely characterized, reinforcing the need for extended pharmacovigilance and real-world studies. Addressing these gaps is critical to achieving sustained neuroprotection and ultimately disease modification across all stages of multiple sclerosis.

## Use of AI tools declaration

The authors declare they have not used Artificial Intelligence (AI) tools in the creation of this article.
